# Effect of early and intensive nutrition care, delivered via telephone or mobile application, on quality of life in people with upper gastrointestinal cancer: study protocol of a randomised controlled trial

**DOI:** 10.1186/s12885-018-4595-z

**Published:** 2018-07-03

**Authors:** Lauren Hanna, Catherine E. Huggins, Kate Furness, Mary Anne Silvers, June Savva, Helena Frawley, Daniel Croagh, Paul Cashin, Liang Low, Judith Bauer, Helen Truby, Terrence Haines

**Affiliations:** 1 Nutrition and Dietetics, Monash Health, Monash Medical Centre, Clayton, VIC Australia; 2 Department of Nutrition, Dietetics and Food, Monash University, Clayton, VIC Australia; 3 Department of Physiotherapy, School of Primary and Allied Health Care, Faculty of Medicine, Nursing and Health Sciences, Monash University, Frankston, VIC 3199 Australia; 4 Centre for Allied Health Research and Education, Cabrini Health, Malvern, VIC 3144 Australia; 5 Upper Gastrointestinal and Hepatobiliary Surgery, Monash Medical Centre, Clayton, VIC Australia; 6 School of Human Movement and Nutrition Sciences, The University of Queensland, St Lucia, Brisbane, QLD Australia; 7 School of Primary and Allied Health Care, Faculty of Medicine, Nursing and Health Sciences, Monash University, Frankston, VIC 3199 Australia; 8 Department of Nutrition, Dietetics and Food, Monash University, Level 1, 264 Ferntree Gully Road, Notting Hill, VIC 3168 Australia

**Keywords:** mHealth, Cost-effectiveness, Malnutrition, Gastric cancer, Oesophageal cancer, Pancreatic cancer, Dietetic intervention

## Abstract

**Background:**

A major challenge for those living with cancers of the upper gastrointestinal tract (oesophagus, stomach and pancreas), is the impact of the disease and treatment on nutritional status and quality of life**.** People with cancer and malnutrition have a greater risk of morbidity and mortality. Nutrition intervention is recommended to commence immediately in those who are malnourished or at risk of malnutrition. Novel cost-effective approaches that can deliver early, pre-hospital nutrition intervention before usual hospital dietetic service is commenced are needed. Linking clinicians and patients via mobile health (mHealth) and wireless technologies is a contemporary solution not yet tested for delivery of nutrition therapy to people with cancer. The aim of this study is to commence nutrition intervention earlier than usual care and evaluate the effects of using the telephone or mHealth for intervention delivery. It is hypothesised that participants allocated to receive the early and intensive pre-hospital dietetic service will have more quality-adjusted life years lived compared with control participants. This study will also demonstrate the feasibility and effectiveness of mHealth for the nutrition management of patients at home undergoing cancer treatment.

**Methods:**

This study is a prospective three-group randomised controlled trial, with a concurrent economic evaluation. The 18 week intervention is provided in addition to usual care and is delivered by two different modes, via telephone (group 1) or via mHealth (group 2), The control group receives usual care alone (group 3). The intervention is an individually tailored, symptom-directed nutritional behavioural management program led by a dietitian. Participants will have at least fortnightly reviews. The primary outcome is quality adjusted life years lived and secondary outcomes include markers of nutritional status. Outcomes will be measured at three, six and 12 months follow up.

**Discussion:**

The findings will provide evidence of a strategy to implement early and intensive nutrition intervention outside the hospital setting that can favourably impact on quality of life and nutritional status. This patient-centred approach is relevant to current health service provision and challenges the current reactive delivery model of care.

**Trial registration:**

27th January 2017 Australian and New Zealand Clinical Trial Registry (ACTRN12617000152325).

## Background

Cancers of the stomach, oesophagus and pancreas are leading causes of cancer deaths in men and women, worldwide [1]. A major challenge for those living with these cancers is the impact of the disease on nutritional status and ultimately, quality of life. Weight loss is often the primary cause of concern to the patient which drives them to initially seek medical advice. Clinically significant weight loss (defined as > 10% body weight) has a reported incidence of 15–69% at time of cancer diagnosis [2–5]. Malnutrition is an inevitable consequence of sustained weight loss, and itself a strong prognostic indicator of mortality [6]. People with cancer and malnutrition have greater risk of post-surgery morbidity [7], debility, compromised immunity, a higher rate of hospital readmission, a longer duration of hospital stay [8], and poorer quality of life [9]. Malnutrition may become established before diagnosis, during or after treatment, and it exists in people with obesity [5].

Clinical management guidelines recommend screening and assessment of malnutrition in cancer patients and advocate for provision of appropriate and effective nutritional therapy, particularly for those where eating may become difficult due to the location of tumours (i.e upper gastrointestinal and head and neck cancers) [10–14]. Evidenced-based interventions to support implementation of these guidelines are lacking. The optimal time for initiating nutrition support is yet to be defined but commencement before malnutrition becomes established is recommended [10]. For patients who are severely malnourished and undergoing active treatment, nutrition intervention should be established immediately [10].

The prevalence of malnutrition at time of diagnosis for people with upper gastrointestinal cancer is as high as 90% in some studies [15]. Early and intensive nutritional intervention can improve nutritional status and Quality of Life (QoL) in both upper gastrointestinal and head and neck cancer patients undergoing radiotherapy [15, 16]. A retrospective review reported on the effect of a nutrition pathway commenced at presentation to a multidisciplinary oesophageal cancer clinic identified that those who received earlier nutritional treatment had significantly greater radiotherapy completion rates (92% vs. 50%), fewer hospital admissions (75% vs. 46%), shorter hospital stays (3.2 days vs 13.5 days) and experienced overall less weight loss (4.2 Kg vs. 8.9 Kg) [8]. A systematic review with meta-analysis evaluated if oral dietary interventions, in people with cancer (any location) who were malnourished or at risk of malnutrition, improved nutritional indices as well as quality of life (QoL) and survival [17]. No effect of oral nutrition intervention was found on mortality (at 6 months). Aspects of QoL were reported to be improved including the global QoL score, and some functional and symptom scale measures. The effects on body weight and other nutrition measures were inconsistent. Overall this review found that there was limited high quality evidence and that more was needed to determine the optimal timing, duration and intensity of nutrition intervention in cancer patients.

To optimise nutritional outcomes, a tailored person-centred approach and frequent review by a dietitian before and during the cancer treatment period is desirable. Novel cost-effective approaches to deliver early, pre-hospital nutrition intervention before current usual care services are commenced, are needed. Linking clinicians and patients via mobile health (mHealth) and wireless technologies is a contemporary solution not yet tested for delivery of nutrition therapy to people with cancer. Research into consumer and practitioner use of, and attitudes towards, integrating technology into healthcare suggests that the right application may be positively accepted by both sets of users [18, 19]. Further, healthcare systems in Europe, Australia and the United States are currently actively encouraging and investing in technological innovations to support available routine care [20, 21].

This present study builds on our pilot study which found that early and intensive dietetic intervention, initiated at the time of diagnosis of gastric or oesophageal cancer was beneficial [15]. The intervention arms included weekly dietetic care delivered over the telephone for 18 weeks (*n* = 10), compared with usual care (*n* = 11). Nutritional status was significantly better amongst intervention group participants than control (adjusting for baseline level) at the first follow-up. This finding is important as it indicates that our early and intensive tele-dietetic intervention may have an impact on malnutrition that develops prior to the commencement of cancer treatment in this patient group. A long term follow-up of this group of patients also found that there may be a slower rate of mortality amongst those exposed to the telephone intervention [22].

In this study two home-based service delivery models will be employed, one via the telephone and the other via a mobile internet enabled communication (i.e mHealth). These service delivery models allow tailored dietary counselling, employing behaviour change techniques and initiation of nutritional supplements if clinically indicated. Telephone and mHealth delivery modes are scalable and they remove the barrier of geography/location of the patient for service delivery thus enabling more equitable access to service. These delivery modes also provide greater flexibility for the patient. The novel mHealth model we propose here also has advantages over telephone in that it tracks progress from both the patient and clinician perspective, for example reporting and documentation of symptoms, weight trajectory and QoL measures; it enables motivational short text messages to be delivered that support and remind the patient of the goals set at low cost; and access to the intervention advice by carers and other health professionals as the information is always available and not reliant on the patient memory of the advice they were given.

There is need to generate more conclusive evidence as to whether early nutrition intervention is effective and feasible in this population. It is also important to determine whether technology-enabled mechanisms for delivery of early nutrition intervention are as effective as telephone-based delivery approaches. We propose a three group randomised controlled trial to address these needs. Specifically, we seek to determine whether an internet-enabled mobile application (App) or intensive telephone counselling intervention can be used to effectively (and cost-effectively) deliver the early nutrition counselling intervention to people with gastric, oesophageal or pancreatic cancer in addition to the usual nutritional care they may later receive. It is hypothesised that participants allocated to receive the early and intensive pre-hospital dietetic service will have more quality-adjusted life years lived compared with control participants.

## Methods

### Study design

**Fig. 1:**
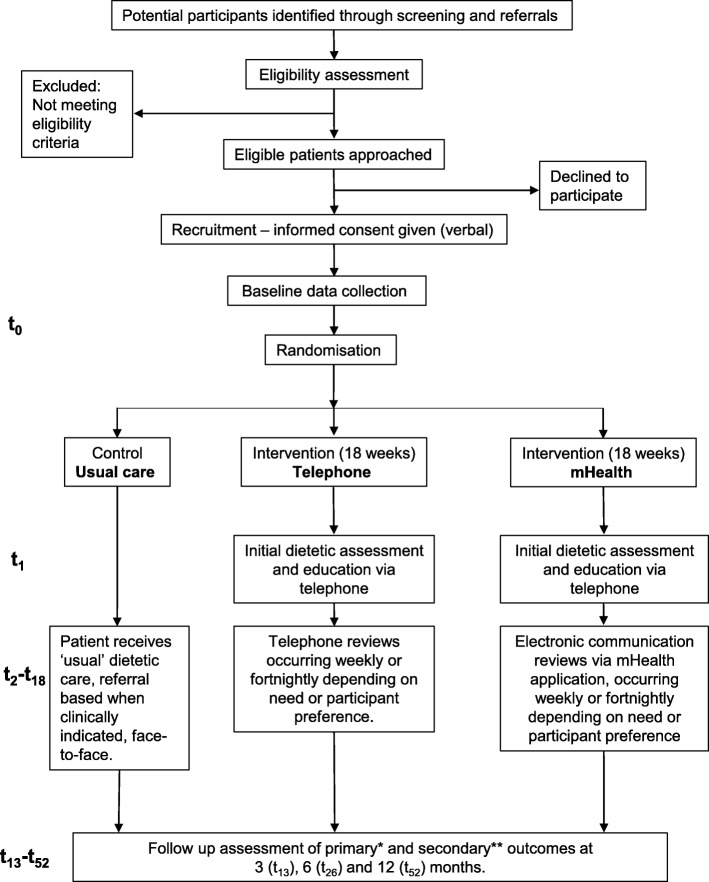
Study flow chart. t_1_ indicates week one of the intervention; t_2_-t_18_ indicates the subsequent weeks of the active intervention; t_52_ indicates the final follow up week and study close. Outcome measures are described in the main text and in summary below: * primary outcome is quality adjusted life years lived determined using the EQ-5D-5 L instrument. ** secondary outcomes include quality of life using the cancer specific EORTC-C30 scale; nutritional status will be measured using the Patient-Generated Subjective Global Assessment (PG-SGA) tool; changes in self-reported body weight; survival over the 12 month follow up period; and where possible waist circumference and hand grip strength using a hand dynamometer

This study is a three-group randomised controlled trial, with a concurrent economic evaluation. Outcomes will be measured at three, six and 12 month follow-up time points. Figure 1 depicts the study design and participant flow.

### Participants and setting

This study will recruit upper gastrointestinal cancer patients who will be receiving surgical and/or chemo/radiotherapy cancer treatment across Health Services (public and private) in Victoria or other states in Australia where the usual care is similar to the usual care services in Victoria. Patients aged > 18 years with suspected or histologically proven diagnosis of primary cancer of the oesophagus, stomach or pancreas will be screened for eligibility to participate. Study sites are listed on the trial registration, and the corresponding author can be contacted to determine if additional sites are recruited.

### Eligibility

Potential participants will be eligible for recruitment up to 4 weeks post histological confirmation of their cancer diagnosis, to allow for early nutrition intervention as compared to current practice. Patients who have already undergone urgent surgical treatment prior to recruitment will be included, however prior commencement of chemotherapy or radiotherapy will deem the patient ineligible, as the commencement of neoadjuvant therapy routinely triggers nutrition intervention as part of ‘usual care.’ Patients who are not considered appropriate for any surgical or chemo/radiotherapy treatment and are therefore receiving ‘end of life care’ will be excluded. Individuals who have insufficient cognition (physician’s judgement) or knowledge of the English language to consent to participation will also be excluded.

### Recruitment

Eligible individuals will be identified via referrals from surgeons, ward dietitians, multidisciplinary team discussions, or screening of weekly outpatient upper gastrointestinal clinic list. Once identified, a member of the patient’s treating team (surgeon, doctor, or cancer care nurse) will be contacted to confirm eligibility and request approval to approach the patient. The recruiter will then make contact with the patient to invite them to participate. This contact will be made in person wherever possible, otherwise via phone. Once an individual gives verbal consent to participate in the study (which is the approved consent process by the Human Research Ethics Committee), baseline data will be collected. The participant’s details will then be provided to the research dietitian, who will arrange for their allocation to a group using sealed, securely stored envelopes. To optimise recruitment, the recruiter attends the weekly Multidisciplinary team meeting at a major tertiary hospital to identify potential participants. The recruiter also sends reminders to Consultants and gives updates of study progress at team meetings to remind Consultants to refer eligible people to be invited to participate in the study. Additional health services across Victoria will be contacted to be study sites to increase access to potential participants if recruitment milestones are not met.

### Randomisation and blinding

Permuted block randomisation with stratification (two groups) will be employed by a biostatistician who is independent of the investigator team. Groups will be stratified based on a malnutrition risk score using the Malnutrition Screening Tool (MST) [23]. The MST is a quick and simple nutrition screening tool based on weight loss and appetite changes. Stratification will be based on a score of < 3 or ≥ 3 as this is routinely used by the supportive cancer care nurse practitioners at the outpatient clinic of Victorian tertiary hospitals for automatic referral to the dietetic outpatient clinic. Computer-generated random numbers are determined in STATA version 14 (StataCorp LP, College Station, Texas, USA). Block sizes will be randomly varied and permuted by the computer software and an initial seed value (a number used to initialize number generation) set for reproducibility. Researchers conducting recruitment, data collection, and data analysis will be blinded to group allocation.

### Usual care

All participants will receive usual care, regardless of group allocation. Standard inpatient dietetic services will be provided subsequent to this using a tailored, symptom-directed approach by a registered dietitian specialising in oncology. This is typically face-to-face counselling with a hospital-based dietitian following medical referral at the commencement of treatment (i.e admission for surgery or chemotherapy commencement). Patients who attend an outpatient service are screened for nutritional risk via MST [23] whereby a score of equal to or greater than three will result in a referral to a hospital dietetic outpatient clinic. The frequency and duration of these sessions will vary on a case-by-case basis. Oral nutritional supplement samples will be supplied if clinically indicated as is consistent with usual care at participating sites.

### Intervention

The intervention is provided in addition to usual care. In this study the same intervention will be delivered by two different modes, via telephone or via mHealth (described in sections below) (Fig. 1). The intervention period will be 18 weeks, commencing as early as possible from the time of diagnosis. Following an initial assessment, weekly or fortnightly reviews will continue throughout the intervention period, as agreed by the dietitian and the participant. Participants who are in hospital at the time of a planned dietetic phone review will not receive their intervention that week, as it is assumed that whilst in hospital they will be receiving dietetic input as required, as part of ‘usual care.’ The intervention will re-commence once the participant has been discharged home, and the intervention timeline will not be suspended.

The intervention will be delivered by a research dietitian with experience in provision of clinical care to this population. The intervention will be delivered according to a Standard Operating Procedure that specifies a list of behaviour change techniques that can be employed through both of the intervention arms (Table 1). The behaviour change techniques specified are drawn from the Behaviour Change Technique Taxonomy (v1) [24]. The dietitian will set goals and instructions on how to perform the behaviour with the participant and provide tailored nutritional recommendations based on the participant’s history and nutrition impact symptoms. In the subsequent review session reviewing of goals will feature and other secondary behaviour techniques can be used if the initial behaviour techniques are not successful (Table 1).

**Table 1:** Example of behaviour change techniques [24] used at the initial assessment (t_1_) and subsequent reviews (t_2_-t_18_)

	Behaviour change technique	Definition [24]	Example	Classification^a^
Initial assessment (t_1_)	Goal setting (behaviour)	Set or agree on a goal defined in terms of the behaviour to be achieved	Set the goal of eating 5 pieces of fruit per day	Routinely Used
	Goal setting (outcome)	Set or agree on a goal defined in terms of a positive outcome of wanted behaviour	Set a weight gain goal (e.g. 0.5 kg over one week) as an outcome of changed eating patterns	Supplementary
	Problem solving	Analyse, or prompt the person to analyse, factors influencing the behaviour and generate or select strategies that include overcoming barriers and/or increasing facilitators	Prompt the patient to identify potential barriers to them drinking a particular supplement (e.g. bad taste) and discuss ways in which they could overcome them (e.g. mix with strawberries)	Supplementary
	Action planning	Prompt detailed planning of performance of the behaviour (must include at least one of context, frequency, duration and intensity). Context may be environmental (physical or social) or internal (physical, emotional or cognitive)	Prompt planning the drinking of a supplement at a particular time (e.g. before work) on certain days of the week	Routinely Used
	Monitoring of behaviours (by self)	Establish a method for the person to monitor and record their behaviour(s) as part of a behaviour change strategy	Ask the person to record daily, in a diary, the amount of food they have eaten	Supplementary
	Monitoring of outcome (by self)	Establish a method for the person to monitor and record the outcome(s) of their behaviour as part of a behaviour change strategy	Ask the person to weigh themselves at the end of each day, over a two week period, and record their daily weight on a graph to increase food intake	Supplementary
	Instruction on how to perform behaviour	Advise or agree on how to perform the behaviour (includes ‘Skills training’)	Demonstrate or describe to person how to prepare thickened fluids	Routinely Used
	Information about antecedents	Provide information about antecedents (e.g. social and environmental situations and events, emotions, cognitions) that reliably predict performance of the behaviour	Discuss how people find it difficult to follow their diet when they attend social events	Supplementary
	Prompts/cues	Introduce or define environmental or social stimulus with the purpose of prompting or cueing the behaviour. The prompt or cue would normally occur at the time or place of performance	Put a sticker on fridge to avoid eating cheesecake	Supplementary
	Graded tasks	Set easy-to-perform tasks, making them increasingly difficult, but achievable, until behaviour is performed	Ask patient to consume supplement once per day the first week, then twice per day the second week.	Supplementary
	Body changes	Alter body structure, functioning or support directly to facilitate behaviour change	Prompt use of dentures to promote food consumption	Supplementary
Review sessions (t_2_-t_18_)^b^	Review goal (behaviour)	Review behaviour goal(s) jointly with the person and consider modifying goal(s) or behaviour change strategy in light of achievement. This may lead to re-setting the same goal, a small change in that goal or setting a new goal instead of (or in addition to) the first, or no change.	Ask if the patient drank the supplement as planned	Routinely Used
	Review goal (outcome)	Review outcome goal(s) jointly with the person and consider modifying goal(s) in light of achievement. This may lead to resetting the same goal, a small change in that goal or setting a new goal instead of, or in addition to the first	Ask if the patient achieved the weight gain goal	Supplementary
	Highlight discrepancy between current and goal (behaviour or outcome)	Draw attention to discrepancies between a person’s current behaviour (in terms of the form, frequency, duration, or intensity of that behaviour) or outcome and the person’s previously set behavioural goals or action plans	Point out that the recorded supplement intake fell short of the goal set	Routinely used
	Monitoring of behaviours (by others, without feedback)	Observe or record behaviour with the person’s knowledge as part of a behaviour change strategy	Have partner observe food intake behaviours and make notes on content and frequency	Supplementary
	Monitoring of behaviours (by others, with feedback)	Monitor and provide informative or evaluative feedback on performance of the behaviour *(*e.g. *form, frequency, duration, intensity)*	Have partner observe food intake behaviours and make notes on content and frequency, inform patient of how many calories they are likely to have ingested per day	Supplementary
	Monitoring of outcome (by others, without feedback)	Observe or record outcomes of behaviour with the person’s knowledge as part of a behaviour change strategy	Weigh the person every two weeks	Supplementary
	Monitoring of outcome (by others, with feedback)	Observe or record outcomes of behaviour with the person’s knowledge and provide informative or evaluative feedback	Inform the person of how much weight they have gained lost following the implementation of a new supplement program	Supplementary
	Social support (unspecified)	Advise on, arrange or provide social support (e.g. from friends, relatives, colleagues,’ buddies’ or staff) or non-contingent praise or reward for performance of the behaviour. It includes encouragement and counselling, but only when it is directed at the behaviour	Arrange for a partner to encourage patient to use supplements	Supplementary
	Social support (practical)	Advise on, arrange, or provide practical help (e.g. from friends, relatives, colleagues, ‘buddies’ or staff) for performance of the behaviour	Ask the partner to mix the supplement with strawberries for the patient	Supplementary
	Consider pros and cons	Advise the person to identify and compare reasons for wanting (pros) and not wanting to (cons) change the behaviour	Advise the person to list and compare the advantages and disadvantages of drinking the supplement	Supplementary

t_1_ indicates week one of the intervention, t_2_-t_18_ indicates the subsequent weeks of the intervention

^a^Behaviour change techniques have been classified as routinely used techniques to be used with all participants, and supplementary techniques that can be optionally be used

^b^These behaviour change techniques are either tied to subsequent sessions because they cannot logically take place during the first session (e.g. review goals), or they are considered to be secondary techniques that one would use if the initial techniques in the Table above had failed

### Community involvement

The study investigators form an advisory group with representatives from the Nutrition, Oncology and Gastroenterology departments at participating sites, local surgical specialists and consumer representatives (i.e cancer survivors). Their feedback was sought on procedures of recruitment, intervention provision, including optimising the mHealth delivery arm and data collection procedures to meet the needs of the participants. Staff working at the study sites were briefed on the study aims, procedure and how this study may impact on their work. Feedback from these consultations was incorporated into the finalised study protocol, prior to trial registration and submission for ethics approval.

### Telephone group

Participants randomised to the telephone group will receive regular phone contact from the research dietitian. An initial assessment will be completed over the phone, with subsequent weekly or fortnightly phone reviews depending on the therapist perception of participant’s needs. Any contact in addition to these scheduled reviews is permissible and will be documented, for example if the participant calls the dietitian with an urgent nutritional issue. Total time spent in conversation with participants will be documented, to allow for a cost analysis between intervention groups (see below).

If a participant is unavailable for a scheduled phone review, the research dietitian will attempt to arrange a more convenient time for the participant. In the event of a second instance that a participant is unavailable in the same week, then no intervention will occur that week. This will be documented, as will the reason for the participant’s unavailability (if known).

### mHealth group

Participants will be provided with a tablet computer (e.g. iPad) and 6 months wireless connectivity if they do not already have access to this equipment. Access to a tablet computer will enable patients to provide fortnightly (or weekly) reports to their dietitian regarding their weight, nutrition impact symptoms (e.g. diarrhoea, mucositis, and nausea) and oral intake so their dietitian can be informed and develop management options prior to their subsequent review via electronic messages. The focus of these consultations is to implement individually tailored, symptom-directed nutritional behavioural management program. Participant engagement in self-monitoring their weight, nutrition impact symptoms, and oral intake using the tablet computer aims to empower and motivate change in dietary behaviours that may be contributing to poor health outcomes [19]. The iPad tool also enables the patient and their carers access to electronic resources that support the nutrition advice.

Communication will be facilitated by a mobile App called MyPace. MyPace has been designed for dietetic practice that supports patient-practitioner relationships [25, 26]. Initial phone contact will occur at baseline to set up the electronic system and complete an initial nutrition assessment. Automated daily reminders will be sent via MyPace to promote behaviour change and adherence. Weight, nutrition impact symptoms (e.g. diarrhoea, mucositis, and nausea) and oral intake will be monitored electronically, and oral nutritional supplements will be recommended as required.

### Adherence

In the event that the research dietitian does not receive any messages or contact from the participant for over a week, the dietitian will make phone contact to confirm that there are no technical issues. Further technical education will be given to the participant as required. The dietitian will not discuss nutritional topics via phone with the participant at any point other than the initial assessment, instead they will encourage the participant to make contact via electronic messages. Total amount of time spent in contact with the participant will be documented, for a cost analysis between intervention groups. This time will be described as nutritional or technical.

### Control group

A usual care, control condition will be used and no additional nutritional advice from the research team will be provided to the participants (Table 2).

**Table 2:** The key points of difference between the intervention groups and usual care control group

1. When nutrition services commence (immediately post-diagnosis versus ~ 12 week delay). 2. The mechanism of access (universal vs referral-based). 3. The mode of service delivery (non face-to-face vs face-to-face). 4. The regularity of service provision (at least fortnightly versus ad hoc, “as required” inpatient basis). 5. The ability to focus on addressing problems before they arise rather than reacting to problems that may already be established.	

### Data collection

The primary method of data collection at each of the four time points (baseline, three, six and 12-months) will be via telephone or face-to-face interview with a blinded researcher. Participants will also be given the option to self-complete and return the questionnaires (described in the outcome sections) to the researcher, via email, stamped and self-addressed envelope, or in person in an inpatient or outpatient setting. These data will be entered into a coded, de-identified spreadsheet. To promote retention, participants will be reminded at each follow-up of the next planned data collection point. It will be explained why these data collection points are important to the study outcomes despite the conclusion of the active intervention period.

### Contamination and co-intervention

Participants in the control arm may seek other sources of dietary advice above what is offered as usual care and obtain oral supplements, which are freely available. Contamination may also be possible (i.e control group participants seek additional dietetic input) if participants enrolled in the study are admitted to the same hospital and allocated beds in the same room as participants in the intervention arms. Participants will be asked to self-report on any nutrition/dietetics information or services they may have accessed during the study period that was additional to the study or usual care services.

### Primary outcome

The primary outcome for this study is quality adjusted life years lived. This will be calculated using the EQ-5D-5 L instrument [27] (as preferred by the UK National Institute for Health and Clinical Excellence [28]) using the area under the curve calculation approach. An area-under-the-curve approach will be used to convert serial EQ-5D-5 L measures into a single estimate of quality-adjusted life years lived following recruitment for each participant. Participants who pass away during the follow-up period will be given a utility value of zero from the date of death onwards. The EQ-5D-5Linstrument is one of the most widely used, generic utility instruments internationally. This instrument has been found to have superior psychometric properties (particularly a lower proportion of participants reporting a “ceiling” score of perfect health and correlation with other measures of health-related quality of life) compared to the EQ-5D-3 L version when used in oncology populations [29] and in non-cancer populations [30]. We will use an EQ-5D-3 L crosswalk value set to generate utility values at each time point before converting to a Quality Adjusted Life Year (QALY) lived estimate [31].

### Secondary outcomes

We will use the EORTC-C30 scale as a secondary measure of health related quality of life [32]. We have included this tool along with the EQ-5D-5 L as it is a disease-specific quality of life instrument that includes items within several domains that are specific to an oncology population. This tool contains 30 items which are separated into domain scores for global health status, physical function, role function, emotional function, cognitive function, social function, fatigue, nausea and vomiting, pain, dyspnoea, insomnia, appetite loss, constipation, diarrhoea, and financial difficulties. This scale has been shown to have high construct validity, ability to differentiate populations with and without cancer, convergent validity with other health state questionnaires, and high test-retest reliability [32, 33].

Nutritional status will be measured using the Patient-Generated Subjective Global Assessment (PG-SGA) tool. This tool is widely used and has been shown to have reliable clinometric properties when used in the oncology population [34, 35]. The first section of the PG-SGA focuses on four areas and when able, is completed by the participant: weight and weight history, food intake history, nutrition impact symptoms, and activities of daily living and function. The researcher will also complete the second section which incorporates specific disease and its relation to nutritional requirements, metabolic demands, and a physical examination to determine loss of subcutaneous fat and muscle wasting if face-to-face data collection is possible.

Other secondary outcome measures include survival over the 12 month follow up period and changes in self-reported body weight. Where possible (if face-to-face data collection is possible) we will measure waist circumference and hand grip strength using a hand dynamometer (Jamar Digital Hand Dynamometer, Lafayette, Indiana, USA) to support our data relating to changes in nutritional status. For the same reason, tissue density from CT scans taken as routine care pre and 3 month post treatment will be analyzed at the 3rd lumbar vertebra to determine body composition (skeletal muscle, visceral and subcutaneous fat) using Slice-O-Matic software (v.5.0; Tomovision, Montreal, Quebec, Canada).

### Economic outcomes

Direct health care costs will be captured by using participant self-report of hospital admissions, hospital records (where accessible), and Medicare and Pharmaceutical Benefits Scheme database extractions. The Medicare and Pharmaceutical Benefits Scheme databases capture all publicly subsidized health care resources consumed in primary care in Australia. The number of days and reasons for hospital admission and outpatient services will be captured from participant self-report and medical records where accessible. Days spent in hospital will be valued using the National Weighted Activity Unit approach which sets prices paid to health services for inpatient and outpatient services provided in Australia (calculator). We will use 2017 as the base year for these calculations.

Productivity costs will be measured using the Productivity Costs Questionnaire (iMTA PCQ). The intent of this measurement is to determine the indirect costs associated with the participant’s health concerns, also known as productivity costs, and whether these are impacted by the intervention. The iMTA PCQ is not disease specific and has been found to be understandable by most of the general population, comprised predominantly of validated questions from previously available instruments [36]. We will use this tool to assess the capacity of participants to perform paid or unpaid work over the trial period, to facilitate the valuation of productivity losses and ensure that the economic evaluation is complete.

### Database extractions

Extraction from hospital databases will be undertaken in an ongoing basis throughout the trial. Extraction from Medical and Pharmaceutical Benefits Scheme databases, and from the Registry of Births, Deaths and Marriages will be completed at the end of the trial.

### Data analysis

The advisory committee will review data cleaning. A mixed methods approach to analysis will comprise a clinical effectiveness study and a concurrent and cost-utility economic evaluation. The primary outcome will be health-related quality of life, converted to quality-adjusted life years lived. Multiple imputation will be used to replace missing individual data points for conducting comparisons in mean QALYs per participant between groups.

### Sample size calculation

A sample size of *n* = 33 participants per group is estimated to attain 80% power to identify a smaller standardised difference (0.70) for comparisons with the control group at the alpha = 0.05 level on the QALY lived outcome. We will therefore recruit *n* = 37 per group to account for potential drop-outs from this study (note – there were zero drop-outs for reasons other than death, from our pilot study with *n* = 21).

### Primary outcome

An area under the curve approach will be undertaken to calculate QALY for each participant. Groups will be compared using regression analyses adjusted for baseline EQ-5D utility values. Analyses will be adjusted for age, gender, baseline PG-SGA category and cancer location (oesophageal, gastric, pancreatic). QALY data from individual participants will be censored at the last available measurement if the participant is lost to follow-up or withdraws.

### Secondary outcomes

Survival over the 12 month follow-up will be compared between groups using Cox proportional hazards analysis with adjustment for age, gender, baseline PG-SGA category and cancer location. Other secondary outcomes will be compared between groups using linear mixed model analyses, adjusting for baseline values of the secondary outcome and age, gender, baseline PG-SGA category and cancer location.

### Economic evaluation analysis

A trial-based, cost-utility analysis will be undertaken to understand what the cost per quality adjusted life year gained is of each intervention group relative to the control group. This economic evaluation will be conducted from the societal perspective over a 12 month follow-up horizon. It will take the form of an incremental cost-utility (cost per quality-adjusted life year gained) analysis. Bootstrap replications of the trial dataset will be used to generate a 95% confidence ellipse surrounding the cost-utility point estimate, and to inform cost-utility acceptability curve analyses. One-way sensitivity analyses will be used to investigate the stability of the main trial finding to variation in key clinical and economic inputs. Exploratory analyses will be undertaken to identify potential intervention interaction effects with age, confidence to use and pre-existing use of iPad/smart phone.

### Advisory and safety committee and data monitoring

An advisory and safety committee is responsible for reviewing milestone attainment and monitoring of recruitment numbers. The committee reviews the overall progress and discusses any unforeseen or adverse events. This committee meets twice per year or more frequently in the event of an adverse event. This committee is chaired by an independent observer of the project (A.S). Committee members include two consumer representatives (i.e cancer survivors, J.W and B.W), and senior clinical investigators not involved in the recruitment, implementation of the protocol (H.T, H.F, M-A.S) and data entry. T.H (principal investigator) and C.E.H (project manager) attend the committee meetings to update on progress, however, they are asked to leave the meeting to permit a closed session so that any issues that may be related to assessing “trial safety and doing no harm” can be done with an impartial view point.

An interim analysis of the data is planned for when 55 participants have completed the 3 month follow up to check that the intervention is “doing no harm”. The intervention data will be coded to preserve blinding of group allocation. Only the Chair (A.S) of the committee will be provided with the results and be unblinded to group allocation to determine the trial is doing no harm. If there is a case to stop the trial early (i.e concern the intervention is doing harm) the Chair will discus the results with the other members of the Advisory committee without T.H and C.E.H.

### Ethics and trial registration

This study has undergone full ethical review by the Monash Health Human Research Ethics Committee and was approved 14th October 2016 (HREC/16/MonH/290). Site-specific authorisation has been granted for all active sites. The trial was registered prospectively on the Australian and New Zealand Clinical Trial Registry (Trial ID: ACTRN12617000152325 27th January 2017) and no amendments have been made to the trial registration. This trial may be spontaneously audited by the overseeing HREC in accordance with their governance procedures.

### Confidentiality

Only investigators who have been named on the ethical approval will have access to the information collected in the study to permit data analysis, interpretation and manuscript/thesis preparation.

### Dissemination

At the completion of the study, it is anticipated that an oral conference presentation will be conducted to communicate the findings from the research to an audience of oncology multidisciplinary health professionals. Two manuscripts will be submitted for publication. They will based on: 1) the main results from the randomised control trial; and 2) cost-effectiveness analysis. The manuscripts will aim to inform researchers, health professionals and policymakers across a number of health fields by adding to the literature field.

The project advisory committee will participate in a review of trial results at the conclusion of the study to help interpret study findings. They will direct formation of a study findings communication plan, identifying relevant stakeholder groups whom they feel should be informed of the study findings and appropriate methods for communicating these findings with them. We will engage with mainstream media (both local and national) upon publication of these results to maximise Australian exposure. We will identify authors of international best-practice guidelines for management of people with upper gastro-intestinal cancer and provide them with copies of our project report and manuscripts arising.

## Discussion

Malnutrition is a common co-morbidity of cancer. The ultimate goal of nutrition intervention is for people with cancer to maintain or improve their nutritional status through adequate nutritional intake. Commencing early nutrition intervention close to time of diagnosis may mean that adequate intake is achieved through oral intake. Tailored interventions for patients undergoing cancer treatments are necessary due to the different treatment modalities, cancer type, tumour location, treatment side effects, food preferences, social circumstances and other co-morbidities these patients may have. This study aims to provide early and tailored nutrition intervention to improve patient outcomes.

With only a small number of randomised controlled trials, most with methodological limitations, there is uncertainty about the effectiveness and timing of nutrition intervention/counselling on clinical outcomes in people with cancers of the upper gastrointestinal tract (oesophagus, stomach and pancreas). Systematic reviews reveal studies examining the effectiveness of dietary interventions for cancer-related malnutrition lack blinding and have small sample size often with heterogeneous cancer types [17, 37]. Most published studies had intervention periods between six to 12 weeks [16, 38–40], with a few extending up to 20 weeks [41–43]. Intervention effects were small but significant for QoL [16, 40] and weight change [39, 42, 43], but these findings were not consistent in all studies [38, 41]. Three studies had a post-intervention follow up at 12 months or beyond and are suggestive of positive association between weight gain and quality of life and longer survival [38, 41, 42].

Studies looking at the effectiveness of oral nutritional supplements are also limited. They are generally of shorter duration (6–10 weeks) than tailored interventions and most did not have a post-intervention follow-up [44–48]. One study investigated oral nutrition supplements with dietary counselling compared with no intervention over a 12 month follow up and reported no difference in changes in quality of life or survival between groups, although this study did find that those surviving beyond 26 weeks had the greatest weight gain within the first 12 weeks of the intervention [38]. The active intervention period in this study was limited to 6 weeks.

Inadequate intensity of the interventions (i.e the frequency of dietetic contact) or limited use of behaviour change techniques [49] may have contributed to the limited effectiveness of nutrition intervention in many studies. Moreover, no study has examined the economic consequences or benefits of these interventions. A strength of the trial we describe in this protocol is that it is commenced early (near the time of diagnosis), it is intensive (i.e at minimum fortnightly reviews) for 18 weeks and is underpinned by behaviour change theory. The evaluation of the economic impact of provision of our tailored nutrition program during cancer treatment is an important and novel contribution to knowledge this study will make.

Studies involving provision of health care interventions using technology-based platforms have great potential to recruit only participants who are not afraid to use these technologies. The problem with this is that there is likely to be a sizeable proportion of the population, particularly amongst older people, who find using technology-based platforms intimidating and a source of increased anxiety [50]. This creates potential for effect sizes drawn from such studies to over-estimate the likely benefit if transferred into real life practice. The three-group design that we are employing, particularly the telephone-based intervention group, is likely to provide incentive for participants to take part who might otherwise have refused consent to participate in this trial if it only had the technology-based intervention group. This means however that people who are reluctant to use technology-based interventions may be allocated to the mHealth group in greater numbers in this trial than if it had just been a two-group trial. Consequently, our three group trial is likely to produce an effect estimate for the mHealth group that is more representative of what might be observed in real life applications.

A key limitation in this study is the length of follow-up we are able to provide being insufficient to generate sufficient statistical power to examine the impact of these interventions on survival. Ideally, this length of follow-up would be extended up to a 5 year period so that the effects of this intervention on both survival and quality-adjusted life years lived could be observed. Previous research has indicated that survival rates in these populations are likely to be below 20% at this time [51], thus this research has great potential to demonstrate benefits on this outcome (if they exist) if further resources can be secured to extend the follow-up of participants in this study out to 5 years.

A limitation of this research related to the interventions being tested is that we are not pursuing a blended online and telephone based intervention delivery approach. The investigators felt it more important to determine whether a technology-based intervention could stand alone as a service delivery model in this population. Asynchronous online interactions with a health professional are attractive as they allow health professionals to be maximally efficient with their work time. There is no time wasted trying to track patients down so that they can talk to them on the phone or speak in person. Instead, feedback from health professionals can be provided at a time convenient to them, making it easier to interact with a greater number of patients in a busy day. Conversely, the strength of the relationship between patient and health professional may be weakened without the direct, synchronous interaction. If there are limitations with this approach, then these limitations need to become apparent through this research.

It is anticipated that successful results from this study could rapidly impact people newly diagnosed with cancers of the upper gastrointestinal tract. Scalable service is possible with either of these service delivery models. The patient-centred approach (delivered at home, at a time convenient to the patient) is relevant to current health service provision and challenges the current reactive delivery model of care.

## Abbreviations

mHealth: Mobile health
MST: Malnutrition screening tool
PG-SGA: Patient-generated subjective global assessment
QALY: Quality adjusted life years
QoL: Quality of life
